# ARHGAP22 Localizes at Endosomes and Regulates Actin Cytoskeleton

**DOI:** 10.1371/journal.pone.0100271

**Published:** 2014-06-16

**Authors:** Mamiko Mori, Koji Saito, Yasutaka Ohta

**Affiliations:** Division of Cell Biology, Department of Biosciences, School of Science, Kitasato University, Kanagawa, Japan; Beatson Institute for Cancer Research Glasgow, United Kingdom

## Abstract

Rho small GTPases control cell morphology and motility through the rearrangement of actin cytoskeleton. We have previously shown that FilGAP, a Rac-specific GAP, binds to the actin-cross-linking protein Filamin A (FLNa) and suppresses Rac-dependent lamellae formation and cell spreading. ARHGAP22 is a member of FilGAP family, and implicated in the regulation of tumor cell motility. However, little is known concerning the cellular localization and mechanism of regulation at the molecular level. Whereas FilGAP binds to FLNa and localizes to lamellae, we found that ARHGAP22 did not bind to FLNa. Forced expression of ARHGAP22 induced enlarged vesicular structures containing the endocytic markers EEA1, Rab5, and Rab11. Moreover, endogenous ARHGAP22 is co-localized with EEA1- and Rab11-positive endosomes but not with trans-Golgi marker TNG46. When constitutively activated Rac Q61L mutant was expressed, ARHGAP22 is co-localized with Rac Q61L at membrane ruffles, suggesting that ARHGAP22 is translocated from endosomes to membrane ruffles to inactivate Rac. Forced expression of ARHGAP22 suppressed lamellae formation and cell spreading. Conversely, knockdown of endogenous ARHGAP22 stimulated cell spreading. Thus, our findings suggest that ARHGAP22 controls cell morphology by inactivating Rac but its localization is not mediated by its interaction with FLNa.

## Introduction

Rho family small GTPases (Rho GTPases) regulate many fundamental cellular processes including cell adhesion, migration, vesicle trafficking, and differentiation [1], [2], [3]. Because Rho GTPases are involved in the control of actin cytoskeleton and cell migration, they are playing an important role in development, immune response, and cancer metastasis [4], [5].

RhoA and Rac1 are well-studied members of Rho GTPases [6]. Rac1 stimulates actin polymerization by activating downstream effectors such as PAK protein kinases and WAVE adaptor proteins. Activation of Rac1 induces formation of lamellae in front of migrating cells and cell spreading on extracellular matrixes (ECMs). RhoA is involved in the generation of contractile force through phosphorylation and activation of myosin II. Thus, RhoA stimulates contraction at the rear of migrating cells and formation of focal adhesion. It is well established that RhoA and Rac1 antagonize each other and define the front-back of moving cells [7].

Rho GTPase functions as a molecular switch in cells. While GTP-bound active form stimulates downstream effectors, hydrolysis of GTP inactivates Rho GTPase. Therefore, they cycle between inactive GDP-bound state and active GTP-bound state. Two classes of proteins mainly regulate this cycle. Guanine nucleotide exchange factors (GEFs) activate Rho GTPase by catalyzing the exchange of GDP for GTP. While GTPase-activating proteins (GAPs) stimulate the intrinsic GTPase activity and inactivate them [8].

ARHGAP22 (also called RhoGAP2 and RhoGAP22) belongs to a family of RhoGAPs that includes FilGAP (ARHGAP24) and ARHGAP25 [9], [10]. The domain structure of ARHGAP22 is similar to that of FilGAP. It contains pleckstrin-homology (PH) domain at its N-terminus, followed by GAP and coiled-coil (CC) domains. Recently, ARHGAP22 has been identified as a key mediator that suppresses Rac1 downstream of RhoA and involved in the amoeboid movement of melanoma cells in 3D environment [5], [11], [12], [13]. Moreover, ARHGAP22 is phosphorylated downstream of Akt and the phosphorylation promotes binding to 14-3-3 protein [14], [15]. We have shown that FilGAP binds to a widely expressed filamentous actin (F-actin) cross-linking protein Filamin A (FLNa) and FLNa binding targets FilGAP to the leading edge of the cell where it antagonizes Rac [9], [10]. In FilGAP, an FLNa-binding site resides to C-terminal to the CC domain [16]. Although ARHGAP22 contains FLNa-binding consensus sequence at its C-terminus [10], it is unclear whether ARHAGAP22 binds to FLNa. Moreover, localization of ARHGAP22 in mammalian cells is unknown.

In this study, we have studied the cellular distribution and function of ARHGAP22. We found that ARHGAP22 does not interact with FLNa. Moreover, we present the evidence that ARHGAP22 localizes at endosomes and is involved in down-regulation of Rac.

## Results

### ARHGAP22 suppresses lamellae formation

Previous study has shown by using RNA interference that ARHGAP22 is involved in regulating the switch between mesenchymal and amoeboid modes of cell migration in 3D environment [11]. Depletion of endogenous ARHGAP22 by RNAi increased GTP-bound Rac and increased the number of mesenchymal melanoma cells [11]. However, it is unclear where ARHGAP22 localizes in cells and how ARHGAP22 regulates actin cytoskeleton. Many growth factors such as EGF induce lamellae through activation of Rac [9]. Therefore, we investigated if ARHGAP22 could function as a RacGAP and suppress lamellae formation induced by EGF.

**Figure 1:**
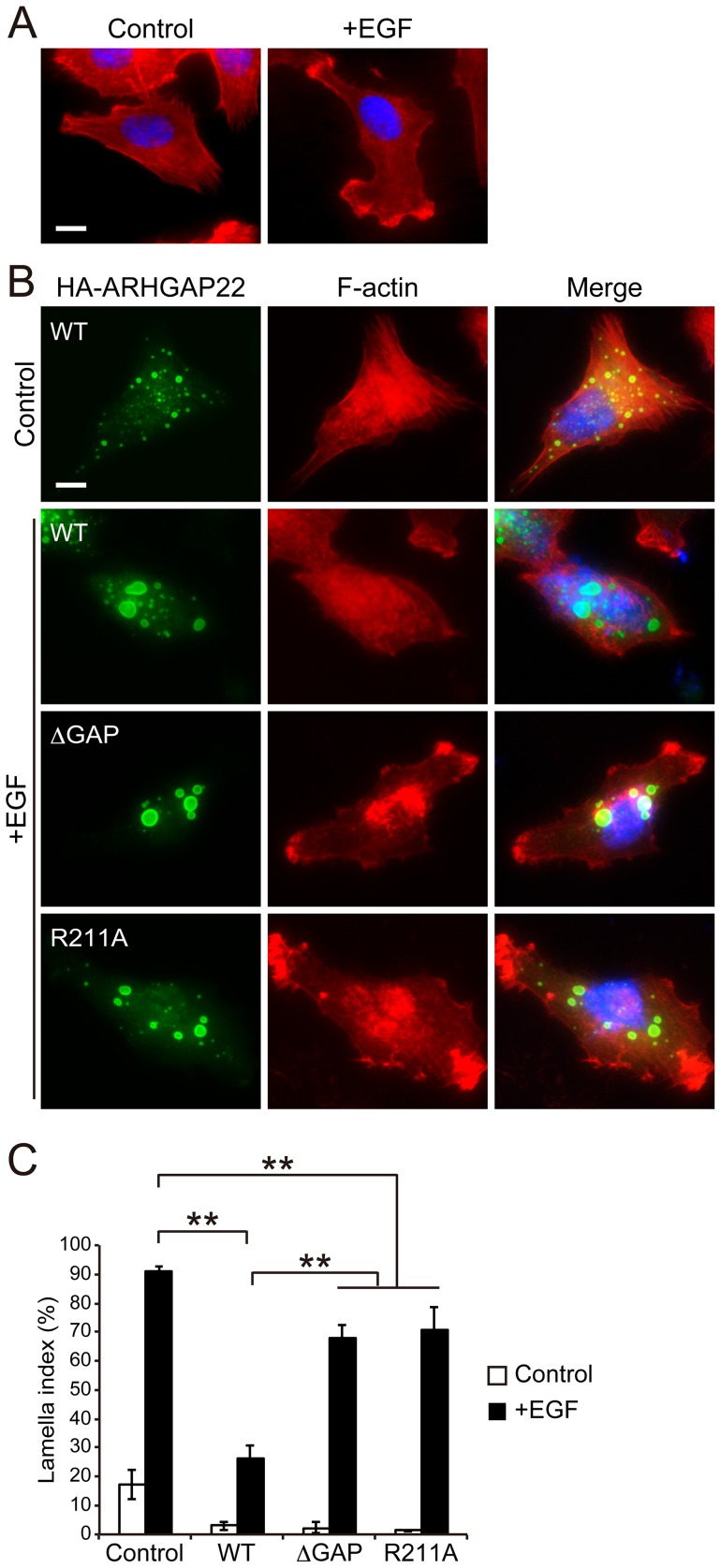
RacGAP activity of ARHGAP22 suppresses lamellae formation. (**A**) EGF-induced lamellae formation. Serum-starved A7 cells were fixed 30 min after the treatment of the cells without (control) or with 50 nM EGF (+EGF) and stained with phalloidin for F-actin (red). The cells were also stained with hoechst 33258 for nuclei (blue). Scale bar, 20 µm. (**B**) A7 cells were either not transfected (control) or transfected with ARHGAP22 constructs (WT, ΔGAP, or R211A) for 5 h and serum-starved. The cells were fixed after treatment without (control) or with 50 nM EGF (+EGF) for 30 min. Representative images of cells stained with anti-HA antibody for HA-ARHGAP22 (green) and phalloidin (red) are shown. Merged fluorescent images are shown. The cells were also stained with hoechst 33258 (blue). Scale bar, 20 µm. (**C**) The percentages of lamellipod-positive cells (n = 100) were calculated, and the data are expressed as the mean ± s.e.m. (N = 3). **, *p*<0.01. Statistical significance was determined by Student's *t*-test.

A7 melanoma cells transfected with ARHGAP22 were stimulated with EGF (50 nM) for 30 min and lamellae formation was analyzed by F-actin staining. More than 90% of control cells produced lamellae (Figure 1A and C), but less than ∼30% of cells produced lamellae when cells were transfected with ARHGAP22 (Figure 1B and C). On the other hand, neither ARHGAP22 lacking its GAP domain (amino acids 163–365; ΔGAP) nor GAP deficient ARHGAP22 (R211A) mutants [14] did not suppress EGF-induced lamellae formation (Figure 1B and C). The GAP-deficient mutants were still capable of inhibiting lamellae formation. The reason is unclear but overexpression of ARHGAP22 could inhibit lamellae formation through RacGAP-independent fashion. We found that ARHGAP22 is mainly localized at punctate structures inside the cells and does not appear to co-localize with actin filaments (Figure 1B and see below). Nonetheless, ARHGAP22 suppresses lamellae formation induced by EGF and its GAP activity is mainly responsible for lamellae suppression.

To confirm if ARHGAP22 functions as a RacGAP, a GST-fusion protein of ARHGAP22 encompassing amino acids 168–365 was prepared and its effect on the intrinsic GTPase-activity of Cdc42, Rac1, and RhoA was determined. The GAP domain of ARHGAP22 stimulated GTPase activity of both Cdc42 and Rac1 but not of RhoA (Figure S1A). Moreover, forced expression of ARHGAP22 effectively reduced the level of GTP-Rac1 but not GTP-Cdc42 and GTP-RhoA as determined by pull-down assay (Figure S1B). These results demonstrate that ARHGAP22 inactivates Rac1 and suppresses lamellae formation.

### ARHGAP22 does not interact with FLNa

**Figure 2:**
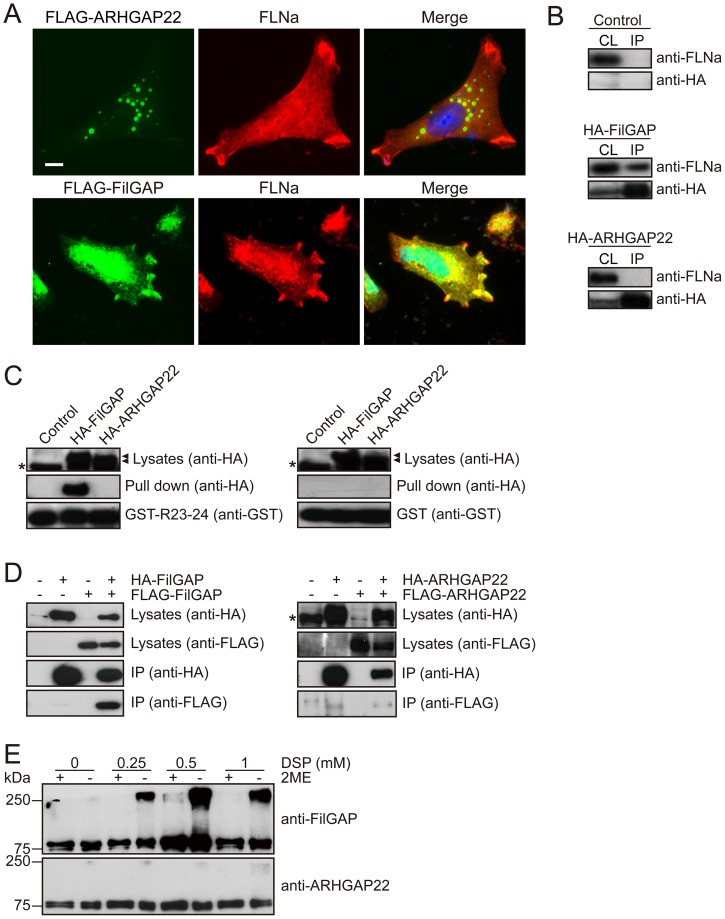
ARHGAP22 does not interact with FLNa. (**A**) A7 cells were transfected with FLAG-ARHGAP22 or FLAG-FilGAP. After 24 h, cells were fixed and ARHGAP22 and FilGAP (green) or FLNa (red) was localized by staining the cells with anti-FLAG and anti-FLNa antibodies. Merged fluorescent images are shown. The cells were also stained with hoechst 33258 for nuclei (blue). Scale bar, 20 µm. (**B**) HEK cells were transfected with HA-ARHGAP22 or HA-FilGAP. HA-ARHGAP22 or HA-FilGAP was immunoprecipitated from cell extracts using anti-HA agarose, and bound proteins were identified by immunoblot using anti-HA and anti-FLNa antibodies. (**C**) HEK cells were transfected with HA-ARHGAP22 or HA-FilGAP. Cell extracts were prepared and then incubated with GST-FLNa-Repeat 23–24 or GST alone, and precipitated with glutathione-Sepharose beads. Bound proteins were analyzed by immunoblot using anti-HA antibody. Asterisks indicate non-specific bands. (**D**) HEK cells were transfected with HA-FilGAP (or HA-ARHGAP22) in the presence or absence of FLAG-FilGAP (or FLAG-ARHGAP22). Then, HA-FilGAP (or HA-ARHGAP22) was immunoprecipitated from cell extracts using anti-HA agarose, and bound proteins were identified by immunoblot using anti-HA and anti-FLAG antibodies. Asterisk indicates a non-specific band. (**E**) HEK cells were transfected with HA-ARHGAP22 or HA-FilGAP. The cells were lysed after treatment with indicated concentrations of DSP, boiled with 1% SDS in the absence or presence of 2-mercaptoethanol (2ME) and analyzed by immunoblot using anti-ARHGAP22 or anti-FilGAP antibody.

As shown in Figure 2A, FilGAP, a close relative of ARHGAP22, localizes at lamellae with FLNa in A7 cells stimulated by EGF. On the other hand, ARHGAP22 localizes at punctate structures at the cytoplasm and does not co-localize with FLNa at lamellae (Figure 2A). We therefore determined if ARHGAP22 binds to FLNa. HEK cells were transfected with HA-tagged full length ARHGAP22 or HA-FilGAP and the proteins were immunoprecipitated from the cell lysates. Although FLNa was co-precipitated with HA-FilGAP, ARHGAP22 failed to precipitate FLNa (Figure 2B). We showed that the C-terminal Repeat 23–24 of FLNa mediates a stable complex with FilGAP [9]. A recombinant GST-Repeats 23–24 construct exhibited strong FilGAP binding activity but the repeats did not bind to ARHGAP22 (Figure 2C).

The C-terminal region of ARHGAP22 contains CC domain similar to FilGAP and has FLNa-binding motif [10], [16]. However, ARHGAP22 does not bind to FLNa *in vitro* and *in vivo* (Figure 2B and C). The high-affinity binding of FilGAP to FLNa is dependent on the dimerization of FilGAP [16]. Therefore, we investigated if ARHGAP22 can dimerize *in vivo*. To investigate whether ARHGAP22 can dimerize in cells similar to FilGAP, we transfected HEK cells with HA-ARHGAP22 and FLAG-ARHGAP22. HA-ARHGAP22 was immunoprecipitated from cell extracts using anti-HA agarose. The immunoprecipitate was separated by SDS-PAGE and immunoblotted for the presence of FLAG- and HA-ARHGAP22 (Figure 2D). Although HA-ARHGAP22 was detectable in the immunoprecipitate as expected, little FLAG-ARHGAP22 was included in the HA-ARHGAP22 immunoprecipitate. On the other hand, FLAG-FilGAP was readily detectable in the HA-FilGAP immunoprecipitate, which was precipitated from co-transfected HEK cells. These results suggest that FilGAP can oligomerize *in vivo*, but ARHGAP22 does not.

We further analyzed if ARHGAP22 could dimerize *in vivo* by using chemical cross-linker DSP (Dithiobis [Succinimidyl propionate]) (Figure 2E). HEK cells transfected with HA-FilGAP were treated with DSP and cell lysates were subjected to SDS-PAGE followed by Western blot for FilGAP. Molecular mass of ∼240 kDa was detected in the presence of DSP. This suggests that FilGAP is present as a multimer in cells. On the other hand, when HA-ARHGAP22 was transfected in HEK cells and treated with DSP, only single band ∼80 kDa was detected and formation of multimer was not detected. These results strongly suggest that ARHGAP22 may present as a monomer *in vivo*.

### Identification of domains mediating subcellular localization of ARHGAP22

**Figure 3:**
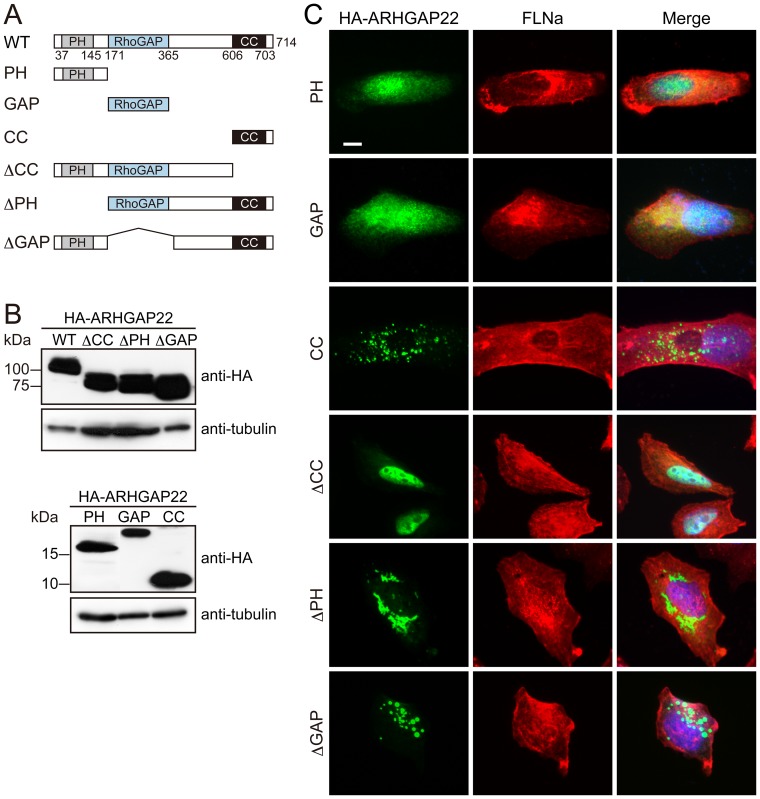
Subcellular distribution of ARHGAP22. (**A**) Schematic diagram of HA-ARHGAP22 constructs. (**B**) Ectopic expression of HA-ARHGAP22 constructs. HEK cells were transfected with HA-ARHGAP22 constructs. HA-ARHGAP22 proteins were analyzed by immunoblot using anti-HA antibody. Tubulin was used as a loading control. (**C**) A7 cells were transfected with HA-ARHGAP22 constructs. After 24 h, the cells were fixed and stained with anti-HA (green) and anti-FLNa (red) antibodies. Merged fluorescent images are shown. The cells were also stained with hoechst 33258 for nuclei (blue). Scale bar, 20 µm.

We delineated respective domains of ARHGAP22 mediating its punctate subcellular localization. We have generated HA-tagged ARHGAP22 constructs (Figure 3A and B), and expressed them in A7 cells. We found that the C-terminal CC domain of ARHGAP22 alone localizes at subcellular punctate structures (Figure 3C). On the other hand, the N-terminal PH domain or GAP domain localizes diffusely in the cell. Moreover, all the ARHGAP22 constructs lacking CC domain failed to localize at punctate structures (Figure 3C). These findings indicate that the CC domain of ARHGAP22 is essential for targeting of ARHGAP22 to punctate structures. Interestingly, we found that ARHGAP22 mutant lacking CC domain (ΔCC) is predominantly localized in nucleus (Figure 3C).

### ARHGAP22 localizes at endosomes

**Figure 4:**
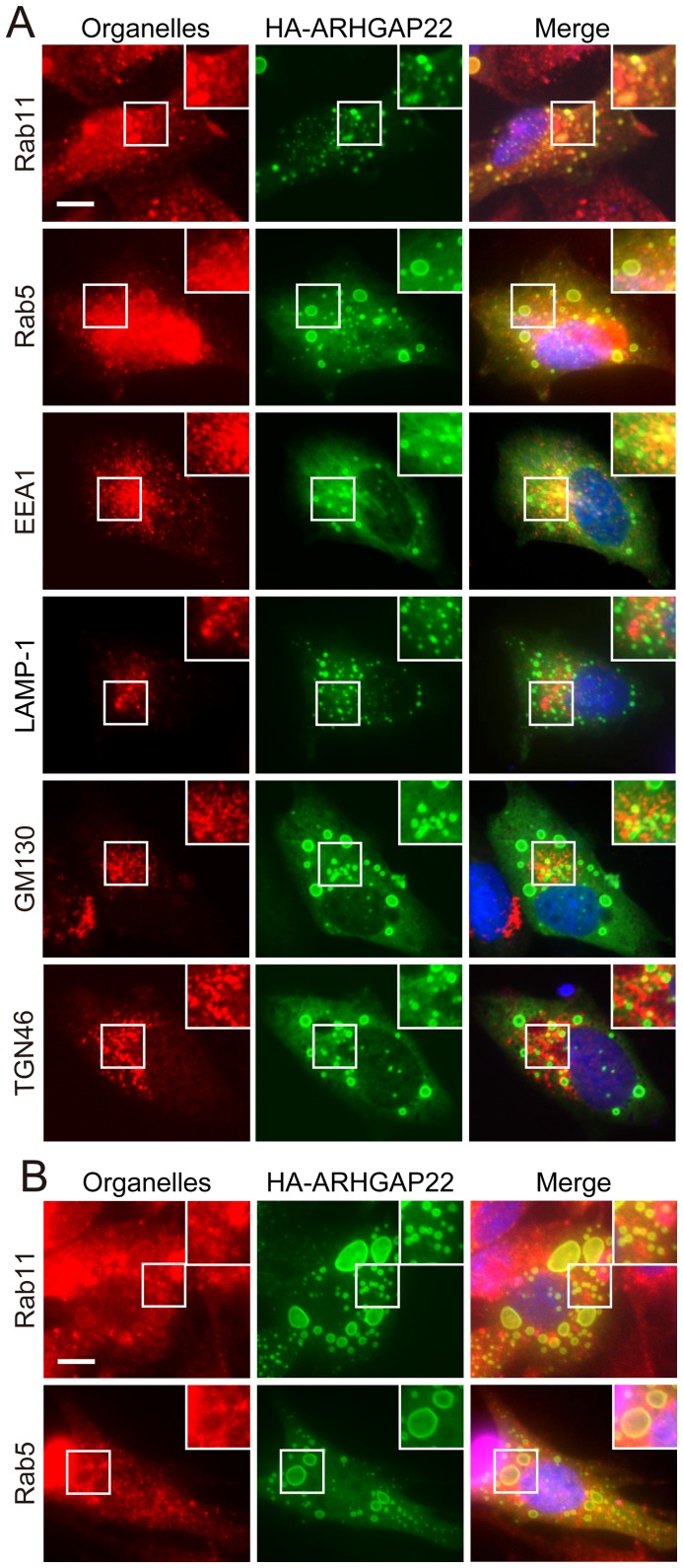
Colocalization of ARHGAP22 with endocytic markers. (**A**) A7 cells were transfected with HA-ARHGAP22. After 24 h, the cells were fixed and stained with anti-HA antibody for HA-ARHGAP22 (green) and antibodies for Rab11, Rab5, EEA1, LAMP-1, GM130, or TGN46 (red). Merged fluorescent images are shown. The cells were also stained with hoechst 33258 for nuclei (blue). Scale bar, 20 µm. Insets show magnification images of the boxed regions. (**B**) C2C12 cells were transfected with HA-ARHGAP22. After 24 h, the cells were fixed and stained with anti-HA antibody for HA-ARHGAP22 (green) and antibodies for Rab11 or Rab5 (green). Merged fluorescent images are shown. The cells were also stained with hoechst 33258 (blue). Scale bar, 20 µm. Insets show magnification images of the boxed regions.

**Figure 5:**
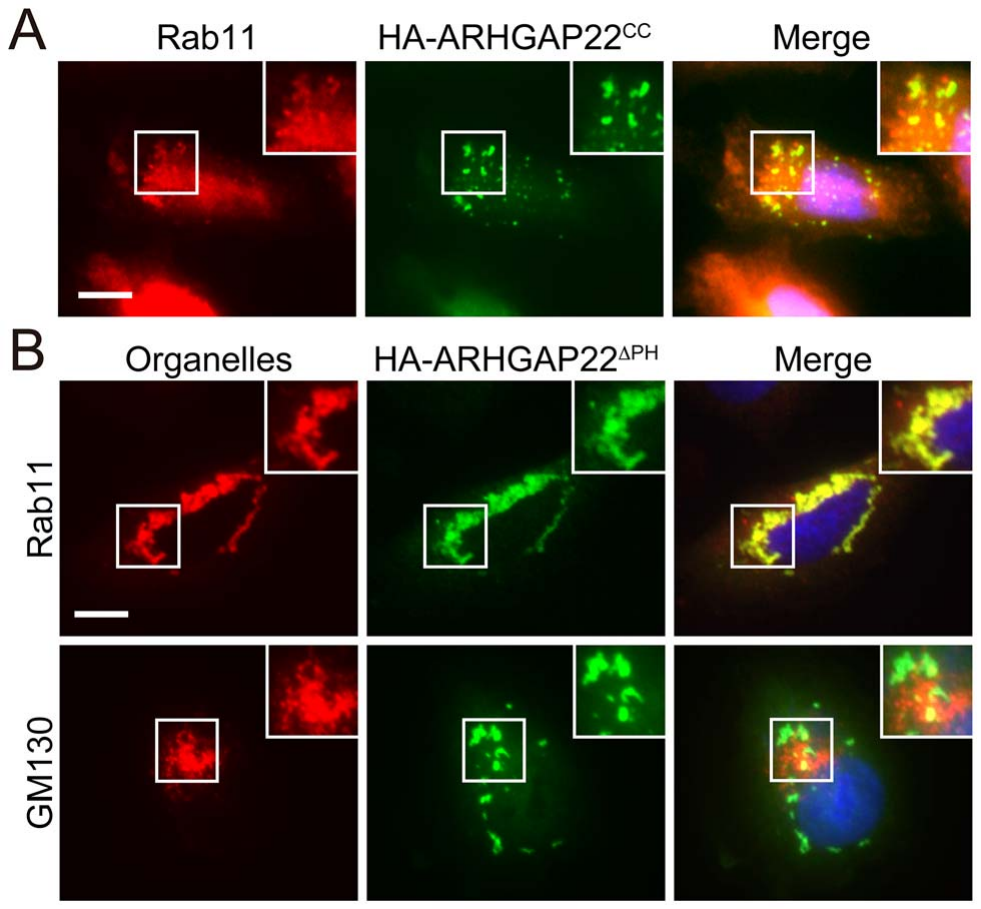
Coiled-coil domain of ARHGAP22 is responsible for targeting to Rab11-positive vesicle structures. (**A**) A7 cells were transfected with HA-tagged coiled-coil (CC) domain of ARHGAP22 (HA-ARHGAP22^CC^). After 24 h, the cells were fixed and stained with anti-HA antibody for HA-ARHGAP22^CC^ (green) and anti-Rab11 antibody (red). Merged fluorescent image is shown. The cells were also stained with hoechst 33258 for nuclei (blue). Scale bar, 20 µm. Inset shows magnification image of the boxed region. (**B**) A7 cells were transfected with HA- ARHGAP22**^Δ^** ^PH^. After 24 h, the cells were fixed and stained with anti-HA for HA- ARHGAP22**^Δ^** ^PH^ (green) and antibodies for GM130 or Rab11 (red). Merge fluorescent images are shown. The cells were also stained with hoechst 33258 (blue). Scale bar, 20 µm. Insets show magnification images of the boxed regions.

To investigate if the punctate structures correspond to any particular organelles in cells, we compared the localization of ARHGAP22 in cells with various organelle markers including: early endosome (anti-EEA1 and anti-Rab5 antibodies), recycling endosome (anti-Rab11 antibody; RE), lysosome (anti-LAMP-1 antibody), Golgi apparatus (anti-GM130 antibody), and trans-Golgi network (anti-TGN46 antibody). We found that punctate structures induced by HA-ARHGAP22 contain endocytic markers EEA1, Rab11, and Rab5 in A7 cells (Figure 4A). Forced expression of HA-ARHGAP22 in mouse myoblast C2C12 cells induced enlarged vesicular structures that also contained Rab11 and Rab5 (Figure 4B). We found that the CC domain of ARHGAP22 alone is sufficient to localize at Rab11-positive structures (Figure 5A). The ARHGAP22 mutant lacking PH domain (ΔPH) showed perinuclear localization (Figure 3C). However, the perinuclear structures still contain Rab11 but do not contain Golgi apparatus marker GM130 (Figure 5B). Therefore, the CC domain of ARHGAP22 may mediate targeting to vesicle structures that contain endosome markers.

**Figure 6:**
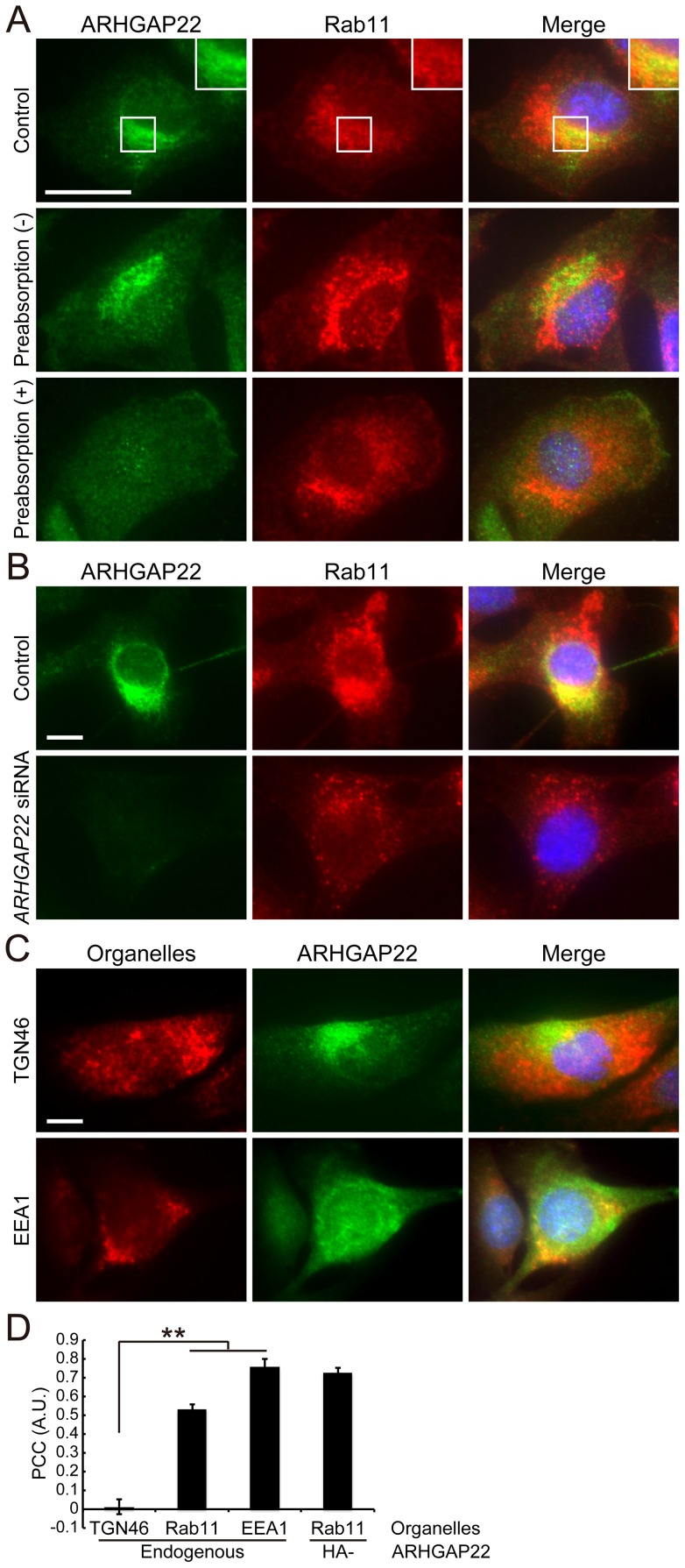
Localization of endogenous ARHGAP22 in C2C12 cells. (**A**) C2C12 cells were fixed and stained with anti-ARHGAP22 (green) or anti-Rab11 (red) antibodies, which was non-treated (control) or preabsorbed with lysates from non-transfected (−) or HA-ARHGAP22-transfected (+) HEK cells. Merged fluorescent images are shown. The cells were also stained with hoechst 33258 for nuclei (blue). Scale bar, 20 µm. Inset shows a magnification image of the boxed region. (**B**) C2C12 cells were treated with control or *ARHGAP22* siRNAs for 48 h and serum-starved. The cells were fixed and stained with anti-ARHGAP22 (green) and anti-Rab11 (red) antibodies. Merged fluorescent images are shown. The cells were also stained with hoechst 33258 (blue). Scale bar, 20 µm. (**C**) C2C12 cells were fixed and stained with anti-ARHGAP22 antibody (green) and antibodies for TGN46 or EEA1 (red). Merged fluorescent images are shown. The cells were also stained with hoechst 33258 (blue). Scale bar, 20 µm. (**D**) Pearson's Colocalization Coefficient (PCC) was calculated by ImageJ (NIH). The data are expressed as the mean ± s.e.m. (N = 3). Ten cells were analyzed for each experiment. **, *p*<0.01. Statistical significance was determined by Student's *t*-test.

To determine the subcellular localization of endogenous ARHGAP22 in mammalian cells, we have prepared rabbit polyclonal antibody directed against amino acid residues 469–485 (RGHRRASSGDRLKDSGS) of human ARHGAP22. The antibody recognized ARHGAP22 but not other family members FilGAP (ARHGAP24) and ARHGAP25 (Figure S2A). The anti-ARHGAP22 antibody also recognized HA-ARHGAP22 protein, which was overexpressed in A7 cells (Figure S2B). By using this antibody, we have analyzed endogenous ARHGAP22 expression in various cell types. Among cell lines tested, we found that mouse C2C12 myoblasts express endogenous ARHGAP22 protein [14]. We therefore determined localization of endogenous ARHGAP22 in C2C12 cells. We found that endogenous ARHGAP22 is localized at punctate structures, which are partly overlapped with Rab11-positive endosomes in C2C12 cells (Figure 6A). Moreover, co-localization of endogenous ARHGAP22 and Rab11 was diminished when the primary antibody was pre-absorbed with the cell lysates expressing HA-ARHGAP22 (Figure 6A). The punctate staining also disappeared after depletion of endogenous ARHGAP22 by siRNA treatment (Figure 6B). The endogenous ARHGAP22 is also partly co-localized with EEA1-positive endosomes but not with trans-Golgi marker TNG46 (Figure 6C and D). Thus, at least in part, endogenous ARHGAP22 seems to localize at Rab11- and EEA1-positive endosomes in C2C12 cells.

### ARHGAP22 suppresses integrin-mediated cell spreading

**Figure 7:**
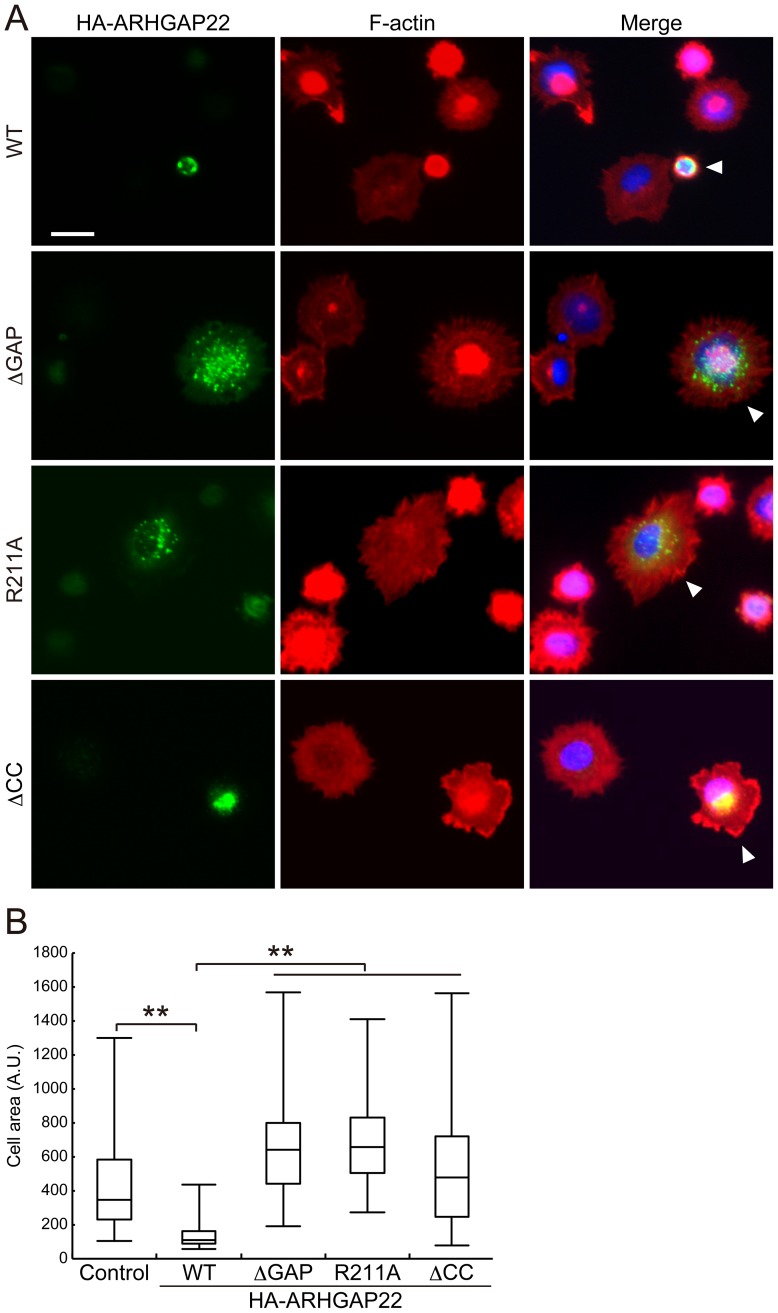
ARHGAP22 suppresses cell spreading. (**A**) A7 cells were transfected with HA-ARHGAP22 constructs (WT, ΔGAP, R211A, or ΔCC,) and serum-starved for 20 h. Quiescent cells were trypsinized and then plated on collagen-coated coverslips and fixed 20 min after plating. The cells were stained with anti-HA antibody for HA-ARHGAP22 (green) and phalloidin for F-actin (red). Merged fluorescent images are shown. The cells were also stained with hoechst 33258 for nuclei (blue). Arrowheads indicate the transfected cells. Scale bar, 20 µm. (**B**) The surface area of spreading cells (n = 100) 20 min after plating were calculated and shown as box and whisker plots. **, *p*<0.01. Statistical significance was determined by Welch's *t*-test.

A7 cells that were plated on collagen-coated dishes adhered within 20 min and then spread circumferentially. Transfection of full-length ARHGAP22 abolished spreading, but ARHGAP22**Δ**GAP and R211A mutants enhanced initial cell spreading (Figure 7A and B), which is consistent with the finding that activation of Rac and Cdc42 by integrin mediates cell spreading [17].

The CC domain of ARHGAP22 mediates targeting of ARHGAP22 to punctate structures (Figure 3 and 5A). Therefore, we have examined if targeting of ARHGAP22 to punctate structures has any role in the control of cell spreading. Forced expression of mutant ARHGAP22 lacking CC domain (ΔCC) failed to suppress cell spreading on collagen. Thus, localization of ARHGAP22 at punctate structures is critical for suppression of cell spreading.

**Figure 8:**
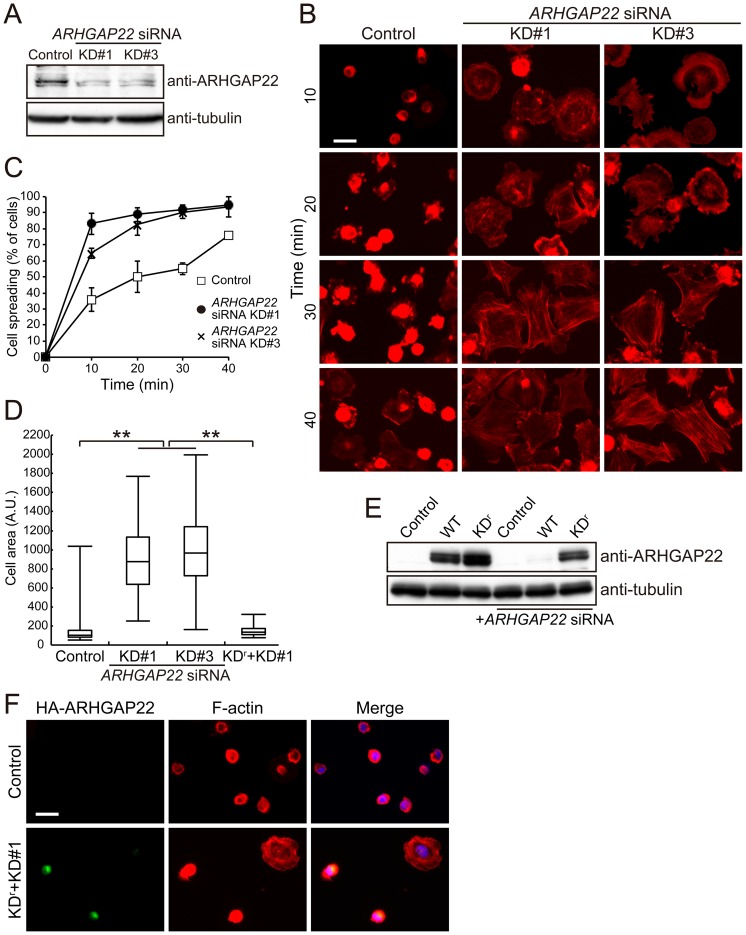
Depletion of ARHGAP22 stimulates cell spreading on fibronectin. (**A**) Immunoblot showing that ARHGAP22 is depleted after 48 h of siRNA treatment of C2C12 cells. ARHGAP22 and tubulin were detected by immunoblot using anti-ARHGAP22 and anti-tubulin antibodies, respectively. (**B**) C2C12 cells were treated with control or *ARHGAP22* siRNAs for 48 h and serum-starved. The cells were trypsinized and then plated on fibronectin-coated coverslips and fixed at 10, 20, 30, and 40 min after plating. The cells were stained with phalloidin for F-actin. Scale bar, 20 µm. (**C**) The percentage of spread cells (n = 200) were calculated and plotted as the mean ± s.e.m. (N = 3). (**D**) The surface area of spreading cells (n = 100) 10 min after plating was calculated and shown as box and whisker plots. **, *p*<0.01. Statistical significance was determined by Welch's *t*-test. (**E**) HEK cells were treated with control or *ARHGAP22* siRNA for 24 h followed by transfection with HA-tagged ARHGAP22 constructs. The cells were cultured for another 24 h. ARHGAP22 and tubulin were analyzed by immunoblot using anti-HA and anti-tubulin antibodies, respectively. (**F**) C2C12 cells were treated with control or *ARHGAP22* siRNA KD#1 for 24 h followed by a transfection with rescue constructs (KD^r^). The cells were cultured for another 24 h and serum-starved. The cells were fixed and stained with anti-HA antibody for HA-KD^r^ (green) and phalloidin (red). Merged fluorescent images are shown. The cells were also stained with hoechst 33258 for nuclei (blue). Scale bar, 20 µm.

To explore the role of endogenous ARHGAP22 in cell spreading, we have transfected C2C12 mouse myoblast cells with small interference RNAs (siRNAs) targeting *ARHGAP22* and spreading on fibronectin was analyzed by F-actin staining. Two-independent siRNAs targeting *ARHGAP22* (KD#1 and KD#3) reduced the expression of endogenous ARHGAP22 in C2C12 cells (Figure 8A), and depletion of ARHGAP22 by these siRNAs promoted much more rapid spreading (Figure 8B and C). The spread area that was occupied by ARHGAP22 RNAi-silenced cells is much bigger than that of control cells 10 min after spreading (Figure 8D).

We introduced 5 silent mutations into the siRNA-targeting sequence of *ARHGAP22* (*KD^r^*) and examined if the spreading on fibronectin that was induced by *ARHGAP22* siRNA could be prevented by *KD^r^*. After two days treatment with ARHGAP22 siRNA, control HA-ARHGAP22 protein was significantly depleted, whereas KD^r^ protein was abundant (Figure 8E). C2C12 cells expressing KD^r^ did not spread on fibronectin in the presence of *ARHGAP22* siRNA (Figure 8F).

### ARHGAP22 co-localizes with constitutively activated Rac at the plasma membrane

**Figure 9:**
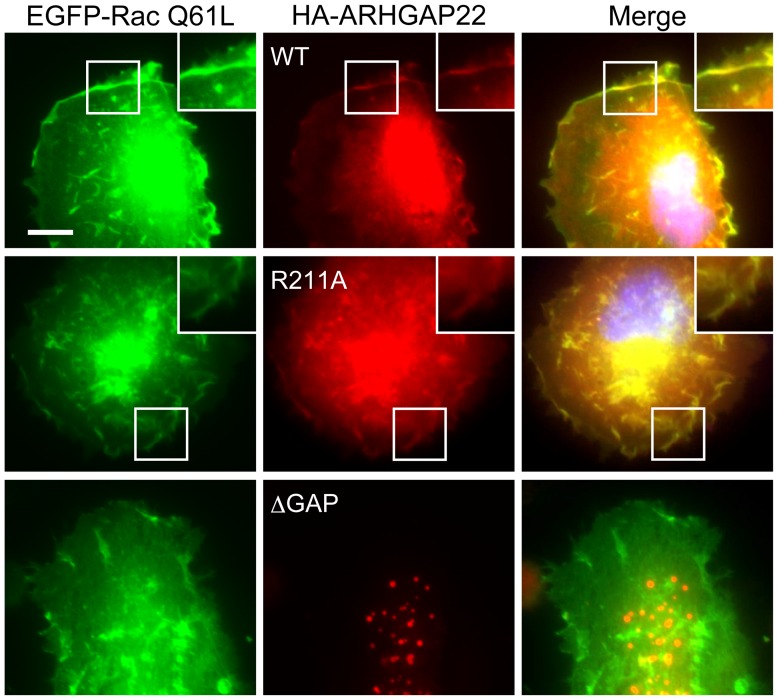
ARHGAP22 co-localizes with constitutively activated Rac at the plasma membrane. A7 cells were transfected with HA-ARHGAP22 constructs (WT, R211A, or ΔGAP) and constitutively activated Rac mutant (EGFP-Rac1 Q61L). After 24 h, the cells were fixed and stained with anti-HA for HA-ARHGAP22 (red). The GFP signal for Rac Q61L (green) was observed in the fixed cells. Merged fluorescent images are shown. The cells were also stained with hoechst 33258 for nuclei (blue). Scale bar, 20 µm. Insets show magnification images of the boxed regions.

To determine if ARHGAP22 could function as a GAP for Rac in cells, we co-expressed ARHGAP22 and constitutively activated mutant Rac (Q61L) in A7 cells. When constitutively activated Rac Q61L mutant was expressed, ARHGAP22 concentrated in sites of membrane ruffles and co-localized with Rac Q61L mutant (Figure 9). Thus, ARHGAP22 could bind to and inactivate Rac at the cell surface although it localizes to the punctate structures in the absence of activated Rac (Figure 4). Targeting of ARHGAP22 to activated Rac at the plasma membrane requires its GAP domain. The GAP deficient ARHGAP22 R211A mutant co-localizes with constitutively activated Rac at the plasma membrane whereas ARHGAP22 mutant lacking its GAP domain (ΔGAP) failed to translocate to the plasma membrane and co-localize with activated Rac Q61L. Thus, GAP domain seems to be a predominant site for interaction with Rac. Forced expression of another constitutively activated Rac G12V mutant induced membrane ruffling and ARHGAP22 was translocated to the ruffles (Figure S1C). On the other hand, translocation of ARHGAP22 to the plasma membrane did not occur when activated mutants of Cdc42 G12V or RhoA G14V were transfected with HA-ARHGAP22 (Figure S1C).

### ARHGAP22 did not affect transferrin receptor-mediated endocytosis

**Figure 10:**
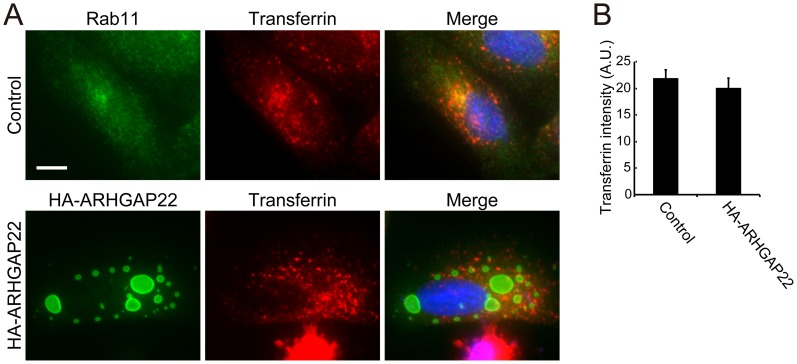
Forced expression of ARHGAP22 does not affect endocytic trafficking of transferrin. A7 cells were either not transfected (control) or transfected with HA-ARHGAP22. After 24 h, the cells were incubated in serum-free growth medium for 1 h. The cells were then incubated with 20 µg/ml Alexa Fluor 568-transferrin at 37°C for 30 min and fixed immediately after incubation. Cells were stained with antibodies for Rab11 or HA-ARHGAP22 (green). Internalized transferrin signals (red) were detected in the fixed cells. Merged fluorescent images are shown. The cells were also stained with hoechst 33258 for nuclei (blue). Scale bar, 20 µm. (**B**) Quantification of the internalized transferrin signal. The transferrin intensity was calculated as fluorescence intensity of Alexa Fluor 568-transferrin per cell divided by the surface area of this cell. The fluorescence intensity and cell area were measured by ImageJ (NIH), and the data are expressed as the mean ± s.e.m. (n = 50 cells). Statistical significance was determined by Student's *t*-test.

Forced expression of ARHGAP22 induced enlarged vesicles that contain endocytic markers Rab11 and Rab5. Therefore, we examined if ARHGAP22 affects receptor-mediated endocytosis using transferrin. After incubation of A7 cells with Alexa Fluor 568-transferrin for 30 min, Alexa Fluor 568-transferrin was endocytosed and partially co-localized with recycling endosome marker Rab11 (Figure 10A). Forced expression of ARHGAP22 induced enlarged vesicles but did not affect incorporation of Alexa Fluor 568-transferrin (Figure 10A and B). The internalized transferrin did not accumulate at the enlarged vesicles (Figure 10A).

## Discussion

In this study, we showed that ARHGAP22 localizes at punctate vesicular structures that contain endocytic markers, and regulates actin cytoskeleton to suppress lamella formation and cell spreading on ECMs.

Although ARHGAP22 has similar domain structure to that of FilGAP, FilGAP localizes along actin filaments and concentrates at lamellae whereas ARHGAP22 primarily localizes at punctate structures that contain endosome markers [9], [18], [19]. Localization of FilGAP at lamellae is mediated by its binding to F-actin cross-linking protein FLNa [9]. FilGAP binds to FLNa through its CC domain. Localization of ARHGAP22 at punctate structures is also dependent on its CC domain. However, we could not detect the binding of ARHGAP22 to FLNa. ARHGAP22 contains consensus sequence for FLNa binding at its CC domain [10]. It has been shown that high affinity binding of FilGAP to FLNa is dependent on dimerization of FilGAP [16]. FLNa is also present as dimer in cells and binding affinity of FilGAP/FLNa interaction is high due to their dimerization [16]. Our study showed that ARHGAP22 seems to present as a monomer in cells. Thus, dimerization of ARHGAP22 may be required for its interaction with FLNa.

It is unclear how ARHGAP22 is targeted to vesicular structures. ARHGAP22 has been shown to interact with several factors. For example, short isoform of ARHGAP22 lacking PH domain (p68RacGAP) binds to transcriptional factor Vezf1 and association induces translocation into nucleus [20]. In neuron, ARHGAP22 (RhoGAP2) is localized to excitatory synapses, and its C-terminal tail interacts with the TIR domain of IL1RAPL1 [21]. Both Vezf1 and IL1RAPL1 are reported to interact with the C-terminus of ARHGAP22. However, they do not seem to be responsible for targeting ARHGAP22 into punctate structures. ARHGAP22 also interacts with β isoform of 14-3-3 [14]. It has been shown that 14-3-3ζ ortholog (PAR-5) plays a role in Rab11-positive recycling endosome positioning and apicobasal cell polarity [22]. However, PH domain of ARHGAP22 has been shown to interact with 14-3-3 [15] and our study demonstrates that CC domain of ARHGAP22 is responsible and sufficient for targeting to Rab11-positive vesicular structures. It is likely, therefore, that the binding of CC domain of ARHGAP22 to punctate structures is mediated by as yet unidentified factors.

Although wild-type ARHGAP22 is mainly localized at vesicular structures, mutant ARHGAP22 lacking CC domain is exclusively localized in nucleus. ARHGAP22 isoform lacking PH domain (p68RacGAP) is also localized in the nucleus when co-expressed with transcription factor Vezf1 [20]. We showed in this study that ARHGAP22 mutant lacking PH domain is localized at perinuclear structures but not in the nucleus. It remains to be determined if ARHGAP22 could function in the nucleus.

Our study suggests that ARHGAP22 may localize at endosomes. First, enlarged vesicles induced by overexpression of ARHGAP22 contain endosome markers (EEA1, Rab5, and Rab11) but not other markers corresponding to Golgi (GM130), lysosome (LAMP-1), and trans-Golgi network (TGN46). Moreover, endogenous ARHGAP22 is present as punctate structures in C2C12 cells and they are partially co-localized with endosome markers (Rab11 and EEA1) but not with trans-Golgi network marker (TGN46). Forced expression of constitutively activated Rab mutants (Rab5 Q79L or Rab22 Q64L) [23], [24], [25] and depletion of Rab7 [26] also induced enlarged endosomes. Therefore, it is possible that ARHGAP22 may affect Rab-dependent endocytic processes through its CC-domain.

Recent studies have shown that membrane trafficking of Rho GTPases plays critical roles in the regulation of cell migration [27]. Rac is endocytosed by Rab5 and activated by RacGEF Tiam1 at the early endosome and recycled back to the plasma membrane through Arf6-dependent pathway [28]. Cdc42 is also activated by αPIX at the early endosome and transported to leading edge by Arf6 to regulate cell polarization [29]. It is therefore possible that ARHGAP22 could function as a RacGAP at endosomes. We showed that forced expression of ARHGAP22 induced co-localization with activated Rac at the plasma membrane. However, little activated Rac was co-localized with ARHGAP22 at endosomes. It is likely that ARHGAP22 may be reserved at endosomes and translocated to the plasma membrane to down-regulate activated Rac.

Although forced expression of ARHGAP22 induced enlarged vesicles in A7 cells, ARHGAP22 did not change the distribution of endocytosed transferrin. The internalized transferrin did not accumulate at the enlarged vesicles induced by ARHGAP22. This is in contrast to Rab5 Q79L- and Rab22-induced vesicles that were accessible to internalized transferrin [23], [26]. Therefore, ARHGAP22 does not seem to have dramatic effects on membrane trafficking. However, regulation of endocytosis and trafficking is receptor-dependent and also relies on experimental conditions. Some studies have reported unaffected recycling of transferrin receptor by Rab5 Q79L [24], while others reported defects in recycling [25]. Rab5 Q79L has been shown to affect EGF receptor recycling [23], [30]. Rab22 has been shown to induce enlargement of early endocytic compartments and recycling of transferrin from Rab22-positive enlarged compartments was blocked [26]. It is well documented that Rab-regulated endocytosis and trafficking of membrane receptors and adhesion molecules are critically involved in the control of cell migration [31], [32]. Further study is required to determine if ARHGAP22 could regulate endocytic pathways of specific membrane receptors and adhesion molecules that are involved in cell migration.

Our present study showed that ARHGAP22 suppresses lamellae formation and cell spreading on ECMs such as fibronectin and collagen. Therefore, ARHGAP22 could regulate actin cytoskeleton in a similar way conducted by FilGAP. Moreover, both FilGAP and ARHGAP22 have been shown to mediate the antagonism between RhoA and Rac1 and AMT (amoeboid-to-mesenchymal transition) in 3D environment [11], [13], [33]. We previously showed that FilGAP specifically inactivates Rac through Rho/ROCK-mediated phosphorylation of FilGAP [9], [33]. It has been shown that depletion of ARHGAP22 inhibited Rho-mediated inactivation of Rac1 and activation of ARHGAP22 is dependent on ROCK/myosin activity [11]. However, it is unclear how ROCK/myosin activity regulates ARHGAP22 at the molecular level. Moreover, our present study demonstrated that cellular localization of ARHGAP22 is completely different from that of FilGAP. ARHGAP22 localizes at endosomes whereas FilGAP is targeted to lamellae. Further study is required to understand how ARHGAP22 is regulated to inactivate Rac downstream of RhoA.

## Materials and Methods

### Plasmids

cDNAs encoding full length *FilGAP* (NM_001025616) and *ARHGAP22* (BC126444) were described previously [9], [13]. cDNAs encoding *ARHGAP22* (wild-type, PH, GAP, CC, ΔPH, ΔGAP, ΔCC, R211A) constructs were inserted into pCMV5-HA or pCMV5-FLAG vector. They were generated as follows; ARHGAP22 was digested with *Eco*RI and *Sph*I to produce PH domain. The GAP domain of ARHGAP22 was generated by PCR. ARHGAP22 was digested with *Eco*RI and *Sal*I to produce CC domain. *ARHGAP22* cDNA lacking PH domain was generated by PCR. *ARHGAP22* cDNA lacking GAP domain was generated by digesting full-length ARHGAP22 with *Xba*I and *Bam*HI. ARHGAP22 lacking CC domain was generated by digesting with *Sma*I and self-ligation. Mutation of R211A of ARHGAP22 construct was generated by introducing point mutations at nucleotide positions 631 and 632 of ARHGAP22 coding sequence using QuikChange site-directed mutagenesis kit (Stratagene, La Jolla, CA). pcDNA3-EGFP-Rac1 Q61L was purchased from Addgene (plasmid ID 12981, Cambridge, MA).

### Cell culture and transfection

HEK293 cells and C2C12 cells were cultured in Dulbecco's modified Eagle's medium (DMEM, Sigma-Aldrich, St. Louis, MO) supplemented with 10% (v/v) fetal bovine serum (FBS) and 50 U/ml penicillin/streptomycin at 37°C. A7 human melanoma cells were cultured in Minimum essential Eagle's medium (MEM, Sigma) supplemented with 2% fetal bovine serum (FBS), 12.5% newborn calf serum, 50 U/ml penicillin/streptomycin and 50 mg/ml geneticin at 37°C. These cells were transfected with plasmid DNA for 24 h using Lipofectamine 2000 (Invitrogen, Carlsbad, CA) or siRNA for 48 h using Lipofectamine RNAimax (Invitrogen) according to the manufacture's instructions.

### Immunoprecipitation

HEK293 cells were transfected with pCMV5-HA-ARHGAP22, pCMV5-FLAG-ARHGAP22, or both. After 24 h, the cells were washed twice with 10 ml of ice-cold Tris-buffered saline (TBS), suspended with 500 µl of lysis buffer containing (20 mM Tris-HCl [pH 7.5], 150 mM NaCl, 1 mM EDTA, and 0.1% NP-40) containing protease inhibitors and incubated on ice for 15 min with shaking. The cell lysates were pre-cleared and supernatant fluids were incubated for 1 h with anti-HA agarose beads (Sigma) at 4°C. Then, the beads were washed three times with lysis buffer, suspended in 60 µl of 1% SDS, boiled, and centrifuged. The supernatants were collected and subjected to SDS-PAGE. Bound-proteins were detected by immunoblot using anti-FLAG or anti-HA antibody.

### Pull-down assay

GST-FLNa-Repeat 23–24 and GST alone were purified from DH5α *E. coli*. Cells transfected with HA-ARHGAP22 or HA-FilGAP were washed with TBS and solubilized in lysis buffer (50 mM Tris-HCl [pH 7.5], 100 mM NaCl, 0.1% NP-40, 5 mM MgCl_2_, 5 mM EGTA, 0.1 mM orthovanadate, 1 mM DTT) containing protease inhibitors. The cell lysates were pre-cleared and incubated with GST-FLNa-Repeat 23–24 in the presence of glutathione-Sepharose 4B (GE Healthcare BioScience, Uppsala, Sweden) for 1 h at 4°C. The glutathione-Sepharose beads were washed four times with lysis buffer and HA-ARHGAP22 or HA-FilGAP was detected by immunoblot using anti-HA antibody.

### Chemical cross-linking with DSP (Dithiobis [succinimidyl propionate])

HEK293 cells were transfected with pCMV5-FLAG-ARHGAP22. After 24 h, the cells were washed twice with 10 ml of Phosphate-buffered saline (PBS) and incubated with 1 mM DSP for 30 min at 25°C. Then, the reaction was stopped by adding 20 mM Tris-HCl (pH 7.4) and incubated for 10 min. The cells were washed and suspended in 1 ml of lysis buffer (20 mM Tris-HCl [pH 7.5], 150 mM NaCl, 1 mM EDTA, and 0.1% NP-40) containing protease inhibitors, and incubated on ice for 15 min with shaking. The cells were homogenized and the cell lysates were prepared by centrifugation at 15,000 rpm for 10 min at 4°C. The lysate samples were boiled for 5 min in the presence or absence of 0.72 M 2-mercaptoethanol and then analyzed by Western blot.

### Immunostaining

Cells were cultured on coverslips (poly-L-lysine coated) transfected with relevant plasmids. After 24 h, cells were washed once with PBS and fixed with 3.7% formaldehyde in PBS for 10 min. The fixed cells were then permeabilized with 0.5% Triton X-100 in PBS for 10 min, then incubated with blocking buffer (10% blocking one [Nakarai Tesque, Kyoto, Japan] in PBS) for 30 min, and immunostained with primary antibodies in blocking buffer for 1 h. Cells were then washed and incubated with Alexa Fluor dye-labeled secondary antibodies (Invitrogen) in blocking buffer for 1 h. For visualization of F-actin and nuclei, cells were stained with Alexa Fluor 568 conjugated-phalloidin and Hoechst 33258, respectively, in PBS for 1 h. After wash with PBS, cells were observed under an Olympus IX81 fluorescence microscope with a 10x, 20x, or 40x objective (Olympus, Tokyo, Japan). Images were acquired by a charge-coupled device camera (ORCA-ER; Hamamatsu photonics, hamamatsu, Japan) and analyzed by MetaMorph software (Molecular Devices, Sunnyvale, CA). Quantification of Pearson's Colocalization Coefficient (PCC) was calculated by Colocalization finder for ImageJ (NIH).

### EGF stimulation

A7 cells were cultured on coverslips (poly-L-lysine coated) transfected with relevant plasmids for 5 h and serum-starved. The cells were fixed after the treatment with 50 nM EGF for 30 min.

### Spreading assay

A7 cells were cultured on 6 cm dish transfected with relevant plasmids for 5 h and serum-starved. After 20 h, the cells were trypsinized and suspended in MEM. The cells were plated on collagen-coated coverslips (50 µg/cm^2^) and fixed 20 min after plating. C2C12 cells were cultured on 6 cm dish treated with *ARHGAP22* siRNAs for 48 h and serum-starved. The cells were trypsinized and suspended in DMEM. The cells were plated on fibronectin-coated coverslips (10 µg/ml) and fixed at 10, 20, 30, and 40 min after plating.

### Transferrin uptake

A7 cells were cultured on coverslips (poly-L-lysine coated) transfected with HA-ARHGAP22. After 24 h, the cells were incubated in serum-free growth medium for 1 h. The cells were then incubated with 20 µg/ml Alexa Fluor 568-conjugated transferrin (Invitrogen) at 37°C for 30 min and fixed immediately after incubation.

### Antibodies

Mouse anti-FLAG (M2) and anti-α-tubulin monoclonal antibodies and rabbit anti-HA and anti-FLAG polyclonal antibodies were purchased from Sigma. Mouse anti-EEA1, anti-GM130, and anti-Rab11 monoclonal antibodies were purchased from BD Biosciences (Bedford, MA). Rabbit anti-TGN46 polyclonal antibody was purchased from Abcam (Cambridge, UK). Mouse anti-LAMP-1 monoclonal and Rabbit anti-Rab5 polyclonal antibodies were purchased from Santa Cruz Biotechnology (Santa Cruz, CA). Mouse anti-HA (12CA5) and anti-FLNa monoclonal antibodies were purchased from Roche Applied Science (Indianapolis, IN) and Millipore (Billerica, MA), respectively. Secondary antibodies conjugated to Alexa Fluor 488 or 568, Alexa Fluor 568-phalloidin (Invitrogen), hoechst 33258 (Dojido laboratories, Kumamoto, Japan) were also purchased from commercial sources. Rabbit anti-FilGAP polyclonal antibody was prepared as described previously [9]. Rabbit anti-ARHGAP22 polyclonal antibody was directed against amino acid residues 469–485 (RGHRRASSGDRLKDSGS) of human ARHGAP22. The peptide was coupled through cysteine at the NH_2_-terminal residue to keyhole limpet hemocyanin (KLH) and was used to raise the antiserum. The antiserum specific to ARHGAP22 was affinity-purified with the immobilized peptide.

### siRNA

siRNA oligonucleotide duplexes targeting human *ARHGAP22* (BC126444) were purchased from Invitrogen. The targeting sequences were as follows: *ARHGAP22*, KD#1 5′-GAUACAUCUGCAAGUUUCUGGAUGA-3′ (nt 902–926) and KD#3 5′-GGAAAUAAAGCUGCGGAACUCUGAA-3′ (nt 2004–2028). For siRNA rescue assay, 5 silent mutations were introduced to the siRNA targeting sequence (nucleotides 902–926). The final mutant was changed into GG ^903^TACATA ^909^TGCAAA ^915^TTC ^918^CTGGAC ^924^GA by PCR. The cells were treated with siRNA for 24 h followed by a transfection with rescue constructs. The cells were cultured for another 24 h and proceed for Western blot or spreading assay.

### Statistical analysis

The statistical significance was accessed by two-tailed unpaired Student's *t*-test or Welch's *t*-test. Differences were considered to be statistically significant at *p* value of <0.01. Error bars (s.e.m.) and *p* values were determined from results of at least three experiments.

## Supporting Information

Figure S1 **ARHGAP22 inactivates Rac1.** (**A**) Recombinant Rac1, Cdc42, RhoA proteins were loaded with [γ^32^-P]GTP and incubated with (filled symbols) or without (open symbols) GST-ARHGAP22-GAP. The γ^32^-P-associated with GTPases was determined at various time points. The data are expressed as the mean of three independent experiments. *, *p*<0.05; **, *p*<0.01. Statistical significance was determined by Student's *t*-test (vs. without GST-ARHGAP22-GAP at each time point). (**B**) HEK cells were transfected with HA-ARHGAP22. Cell lysates were incubated with GST-PAK1-CRIB for Rac1 and Cdc42 or GST-Rhotekin-RBD for RhoA that was immobilized on glutathione-Sepharose beads. The amount of Rho GTPases in cell lysates before pull-down and GTP-bound Rho GTPases was detected by immunoblotting using anti-Rac1, anti-Cdc42, or RhoA antibody. Asterisks indicate nonspecific bands. (**C**) A7 cells were transfected with HA-ARHGAP22 and constitutively activated Rac (Myc-Rac G12V), Cdc42 (FLAG-Cdc42 G12V), or RhoA (Myc-RhoA G14V) mutants. After 24 h, the cells were fixed and stained with anti-HA (green) and antibodies for Myc or FLAG (red). Merged fluorescent images are shown. The cells were also stained with hoechst 33258 (blue). Scale bar, 20 µm. Inset shows magnification image of the boxed region.
(TIF)

Figure S2 **Production of antibodies against ARHGAP22.** (**A**) Specificity of anti-ARHGAP22 antibody was shown by immunoblotting from HEK293 cells transfected with a control plasmid (pCMV5-HA) or pCMV5-HA plasmids encoding human *FilGAP*, *ARHGAP22*, or *ARHGAP25*. The HA-epitope and tubulin (loading control) were also detected by immunoblotting using anti-HA and anti-tubulin antibodies, respectively. Arrowheads and asterisks indicate HA-tagged proteins and non-specific bands, respectively. (**B**) A7 cells were transfected with HA-ARHGAP22. After 24 h, the cells were fixed and stained with anti-ARHGAP22 (green) and anti-Rab11 (red) antibodies. Merged fluorescent image is shown. The cells were also stained with hoechst 33258 for nuclei (blue). Scale bar, 20 µm.
(TIF)
