# Supplementary figures and images for: ARHGAP22 Localizes at Endosomes and Regulates Actin Cytoskeleton
Source: PLoS One. 2014 Jun 16;9(6):e100271. doi: 10.1371/journal.pone.0100271 (PMC4059726; doi:10.1371/journal.pone.0100271)

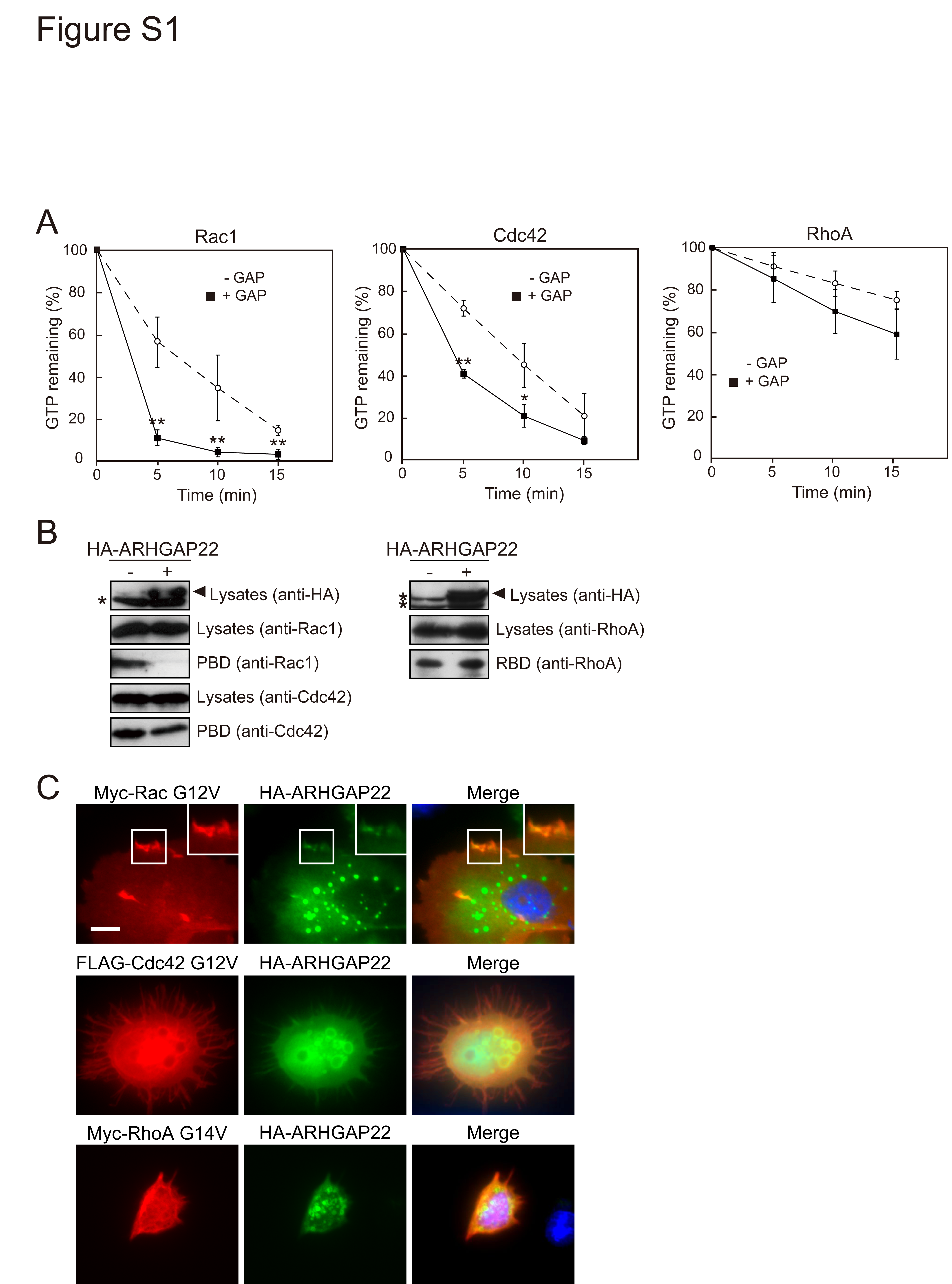

Supplement: Figure S1 — ARHGAP22 inactivates Rac1. (A) Recombinant Rac1, Cdc42, RhoA proteins were loaded with [γ32-P]GTP and incubated with (filled symbols) or without (open symbols) GST-ARHGAP22-GAP. The γ32-P-associated with GTPases was determined at various time points. The data are expressed as the mean of three independent experiments. *, p<0.05; **, p<0.01. Statistical significance was determined by Student's t-test (vs. without GST-ARHGAP22-GAP at each time point). (B) HEK cells were transfected with HA-ARHGAP22. Cell lysates were incubated with GST-PAK1-CRIB for Rac1 and Cdc42 or GST-Rhotekin-RBD for RhoA that was immobilized on glutathione-Sepharose beads. The amount of Rho GTPases in cell lysates before pull-down and GTP-bound Rho GTPases was detected by immunoblotting using anti-Rac1, anti-Cdc42, or RhoA antibody. Asterisks indicate nonspecific bands. (C) A7 cells were transfected with HA-ARHGAP22 and constitutively activated Rac (Myc-Rac G12V), Cdc42 (FLAG-Cdc42 G12V), or RhoA (Myc-RhoA G14V) mutants. After 24 h, the cells were fixed and stained with anti-HA (green) and antibodies for Myc or FLAG (red). Merged fluorescent images are shown. The cells were also stained with hoechst 33258 (blue). Scale bar, 20 µm. Inset shows magnification image of the boxed region. (TIF) [file pone.0100271.s001.tif]

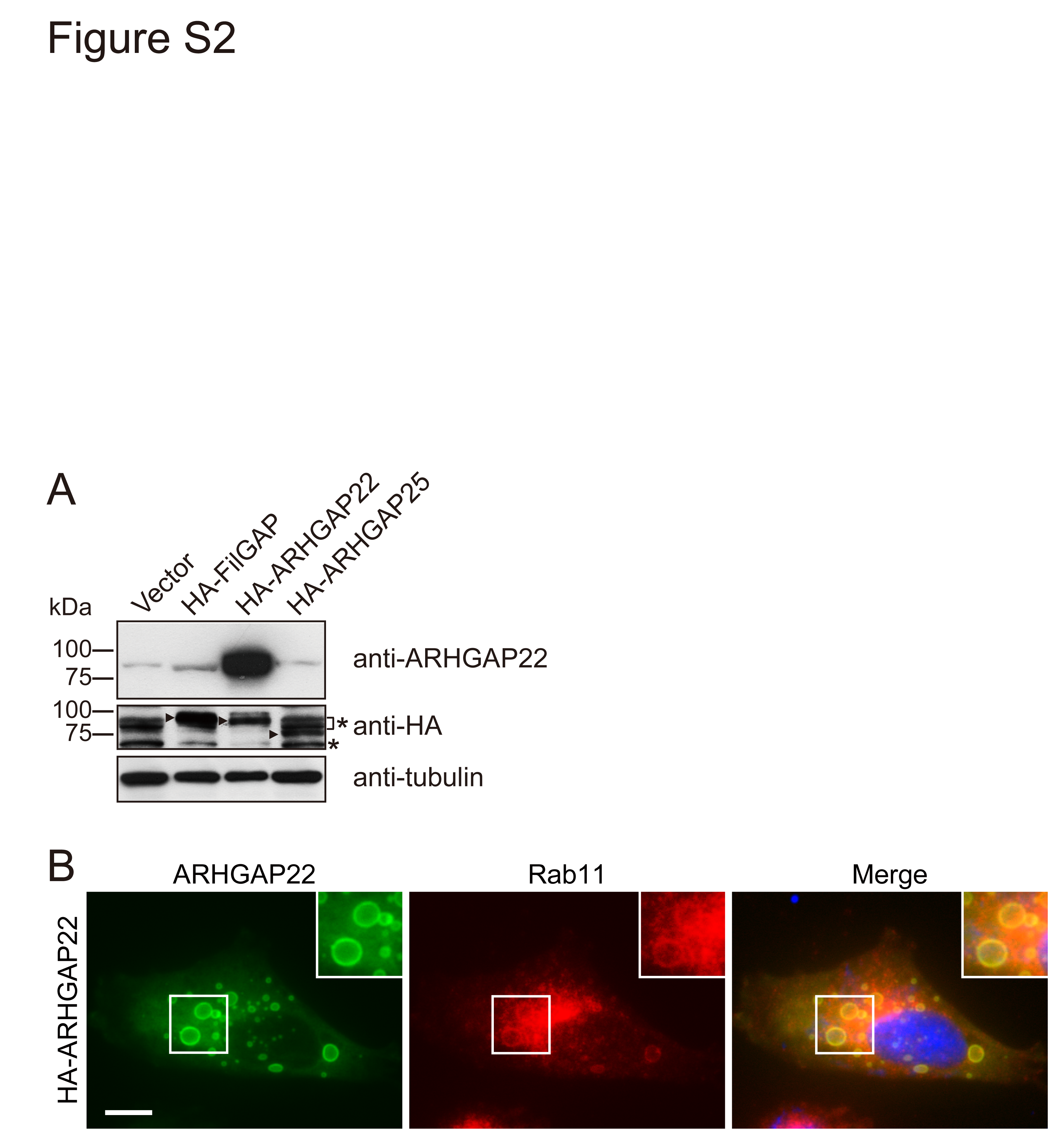

Supplement: Figure S2 — Production of antibodies against ARHGAP22. (A) Specificity of anti-ARHGAP22 antibody was shown by immunoblotting from HEK293 cells transfected with a control plasmid (pCMV5-HA) or pCMV5-HA plasmids encoding human FilGAP, ARHGAP22, or ARHGAP25. The HA-epitope and tubulin (loading control) were also detected by immunoblotting using anti-HA and anti-tubulin antibodies, respectively. Arrowheads and asterisks indicate HA-tagged proteins and non-specific bands, respectively. (B) A7 cells were transfected with HA-ARHGAP22. After 24 h, the cells were fixed and stained with anti-ARHGAP22 (green) and anti-Rab11 (red) antibodies. Merged fluorescent image is shown. The cells were also stained with hoechst 33258 for nuclei (blue). Scale bar, 20 µm. (TIF) [file pone.0100271.s002.tif]
